# Molecular dynamic simulations reveal suboptimal binding of salbutamol in T164I variant of β2 adrenergic receptor

**DOI:** 10.1371/journal.pone.0186666

**Published:** 2017-10-20

**Authors:** Srinivas Bandaru, Mallika Alvala, Anuraj Nayarisseri, Saphy Sharda, Himshikha Goud, Hema Prasad Mundluru, Sanjeev Kumar Singh

**Affiliations:** 1 Institute of Genetics and Hospital for Genetic Diseases, Osmania University, Hyderabad, India; 2 Molecular Modeling Lab, Department of Medicinal Chemistry, National Institute of Pharmaceutical Education and Research, Hyderabad, India; 3 In Silico Research Laboratory, Eminent Biosciences, Indore, Madhya Pradesh, India; 4 Bioinformatics Research Laboratory, LeGene Biosciences Private Limited, Indore, Madhya Pradesh, India; 5 Computer Aided Drug Designing and Molecular Modeling Lab, Department of Bioinformatics, Alagappa University, Karaikudi, Tamil Nadu, India; Jamia Millia Islamia, INDIA

## Abstract

The natural variant C491T (rs1800088) in ADRB2 gene substitutes Threonine to Isoleucine at 164^th^ position in β2AR and results in receptor sequestration and altered binding of agonists. Present investigation pursues to identify the effect of T164I variation on function and structure of β2AR through systematic computational approaches. The study, in addition, addresses altered binding of salbutamol in T164I variant through molecular dynamic simulations. Methods involving changes in free energy, solvent accessibility surface area, root mean square deviations and analysis of binding cavity revealed structural perturbations in receptor to incur upon T164I substitution. For comprehensive understanding of receptor upon substitution, OPLS force field aided molecular dynamic simulations were performed for 10 ns. Simulations revealed massive structural departure for T164I β2AR variant from the native state along with considerably higher root mean square fluctuations of residues near the cavity. Affinity prediction by molecular docking showed two folds reduced affinity of salbutamol in T164I variant. To validate the credibility docking results, simulations for ligand-receptor complex were performed which demonstrated unstable salbutamol-T164I β2AR complex formation. Further, analysis of interactions in course of simulations revealed reduced ligand-receptor interactions of salbutamol in T164I variant. Taken together, studies herein provide structural rationales for suboptimal binding of salbutamol in T164I variant through integrated molecular modeling approaches.

## Introduction

Inhaled β2 agonists form first line treatment stratagem for the management of intermittent symptoms to severe exacerbations in asthma and chronic obstructive pulmonary disease. With their rapid onset of action along with minimal side-effects [1, 2], inhaled β2 agonists enhance lung function and improve symptoms of shortness of breath [3–5]. In fact, international guidelines of asthma management itself recommend rapid-onset inhaled β2AR agonists alone for symptomatic relief from airway constriction [6–11].

In the early 19^th^ century, bronchodilators such as epinephrine, isoproterenol were introduced into clinical practice with promise of “standard-of-care sympathomimetics” for acute bronchoconstriction [12]. Although, anticipated to be perfect bronchodilators, epinephrine, isoproterenol and long been used ephedrine were however short duration relievers with slow onset of action which fell short to achieve desired therapeutic effect [13–16]. In addition, these drugs suffered various pharmacodynamic setbacks including low potency, high metabolic instability and administration often followed parenteral routes [17, 18]. With regular use, epinephrine, isoproterenol predisposed patients to cardiac dysarhythmia which was later attributed to its non-selective targeting for β1AR and β2AR [19]. The expression of β1ARs and β2-ARs is contrasting in myocardial and airway epithelium, while β1ARs are expressed in myocardial cells, diminished expression is observed in airway epithelium and *vice versa* [20]. Apparently, expression profiles of βARs therefore clearly explain cardiac dysarhythmias brought about by non-selective nature of agonists like epinephrine or isoproterenol. In fact, investigations following slight rise in mortality rates in 1940s and early 1950s linked regular use of non-selective β2 agonists with myocardial complications [21]. While the quest for developing structure based agonist selectively targeting β2AR was in the demand, Sir David Jack and colleagues at Allen and Hanburys (now part of GlaxoSmithKline) introduced salbutamol [22]. At recommended clinical doses salbutamol has negligible α-AR activity and shows substantial selectivity between beta adrenergic receptor isoforms [23]. The discovery of salbutamol was certainly a breakthrough as it revolutionized asthma management within months of appearing in the market. In fact salbutamol still remains first line of treatment for airway obstruction globally since its discovery past 52 years [24].

Bronchodilation events follow an intricate cascade of GPCR activated cell signaling process. Persistent activation of β2AR is achieved through binding of β2 agonist and a G protein at opposite ends of the receptor relative to the lipid bilayer [25]. Binding of agonist activates adenyl cyclase and increases cAMP levels which in turn phosphorylate downstream protein modulators by enhancing of protein kinase A (PKA) activity [26]. The overall agonist induced activation decreases intracellular Ca^2+^ levels prompting hyperpolarization of the airway smooth muscle for dilation [27, 28].

Latest genetic approaches like GWAS (Genome Wide Association Studies) identified almost 30 genes with pathological modulation of respiratory diseases. Of particular relevance, alterations in the ADRB2 gene surfaced as a significant determinant in modulating β2 agonist response in mild to severe airway obstruction [29–32]. In addition, recombinant expression and site-directed mutagenesis investigations have shown significant impact on receptor function leading to altered response to β2 agonists [33]. Interestingly, major evidence associating ADRB2 variants with functioning of the receptor comes from C→T base exchange (rs1800088) at the 491 position in ADRB2 gene that substitutes Threonine to Isoleucine at 164^th^ position in the β2 adrenergic receptor (β2AR). The consequence of variation is observed as diminished ligand-receptor interactions followed by depressed coupling of β2AR to adenylcyclases wherein dose-response binding curve shifts to right indicating the low affinity of the agonists to the T164I variant [34]. In addition, pioneering studies by Strader *et al* in 1989 suggested that Threonine to Isoleucine substitution in β2AR significantly decreases spontaneous toggling to the activated state and reduces agonist stabilized activation resulting in declined capacity of the receptor to interact with β2 agonists [35].

Deemed to the imperative role of β2AR pharmacogenetics, in our recent studies we reported significant association of β2AR T164I polymorphism with salbutamol refractoriness in asthmatics presenting with considerable decline in lung function volumes [36]. As mentioned afore, studies have also suggested suboptimal binding affinity of agonists to T164I variant; we therefore hypothesized that the refractoriness observed in asthmatics may as well be attributed to reduced interactions of salbutamol in patients homozygous for polymorphic T164I β2AR. To testify and prove our hypothesis, the present study is pursued to put forth the molecular rationales of suboptimal binding of salbutamol in T164I variant employing exhaustive and integrated molecular modeling approaches involving molecular dynamic simulations.

## Materials and methods

### Protein optimization, modeling of T164I β 2AR variant and ligand preparation

The crystal structure of β2AR was retrieved from Protein Data Bank [PDB entry 3NY8] with a resolution of 2.84 Å [37]. The structure was optimized and prepared by assigning bond orders; charges, hybridizations and explicit hydrogen were added if missing. The missing side chains and loops in the structure were filled using prime module of Schrödinger suite 2015. The structure was further reviewed by deleting crystal water molecules and hetero atoms except for the inverse agonist (PDB ID: JRZ—(2S,3S)-1-[(7-methyl-2,3-dihydro-1H-inden-4-yl)oxy]-3-[(1-methylethyl)amino]butan-2- ol) present at the active site of the crystal structure. Prior to energy minimization, the structure was stabilized at the physiological pH of 7.0. The resulting structure was refined by energy minimization using Optimized Potential for Liquid Simulation (OPLS—2005) algorithm with complete structure converging to root mean square deviation (RMSD) of 0.30 [38]. A complete method of protein preparation, modification and refinement was performed by protein preparation wizard (PrepWiz) module of Schrödinger suite 2015. The T164I variant structure of β2AR was generated by substituting Threonine to Isoleucine at 164^th^ position in the backbone using Pymol Schrödinger LLC 2010. The structure obtained from mutation was further optimized, and energy minimized as mentioned afore. Ligand preparation for (R)-(−)-enantiomer (biologically active form) of salbutamol was performed in Ligprep module of Schrödinger suite 2015.

### Determination of vulnerability, functional and structural consequences of variation

The vulnerability of SNP (rs1800088) C→T in the ADRB2 gene (T164I) was evaluated with mutation assessor modules *viz*, SIFT [39], Polyphen 2 [40]. SIFT—a vector based method calculates tolerance index (score ranging from 0 to 1) for a particular residue substitution based on structural and functional parameters using multiple sequence alignments against homologous amino acid sequences. PolyPhen 2 is also a vector based method that calculates the pathogenic potential of nsSNPS that considers comparison of sequences, 3D protein structures and residue contacts, returning results as PSIC score (higher PSIC score reflects higher the functional effect on protein and vice versa). In order to obtain consistent and unbiased predictions, along with SIFT and Polyphen, efficient prediction programs such as MAPP, PhD-SNP, SNAP and Panther integrated in Meta-SNP [41] and Predict SNP [42] web interfaces were also used to determine the vulnerability of variation.

The free energy changes incurred due to T164I substitution was calculated by vector based machine learning methods like I-Mutant 3.0 [43] Strum [44] and Site Directed Mutator (SDM) [45] programs. The support vector machine supervised I-Mutant 3.0 uses a dataset derived from ProTherm [43]. I-Mutant predictor efficiently evaluates the stability change upon single site mutation from the protein structure or protein sequence and returns Gibbs energy changes as the function of ΔΔ*G* value. STRUM’s robust and accurate method in determining fold stability upon residue mutations lies in gradient boosting regression approach which trains Gibbs energy changes at different levels of sequence and structural properties. STRUM uses combination of sequence profiles with low-resolution structure models from protein structure prediction which makes it applicable to various protein sequences, including those without experimental structures. Changes in solvent accessibility surface area, secondary structure and fold stability in the protein structure upon amino acid mutation was analyzed employing Site Directed Mutator (SDM). SDM uses statistical potential energy functions which analyzes the effect of single nucleotide polymorphisms on protein function and predicts malfunctioning probability in terms of protein stability.

### Ligand-receptor affinity prediction by molecular docking

Glide version 6.1 (Grid Based Ligand Docking with Energetics) program of Schrödinger suite (Schrödinger, LLC, New York, NY, 2013. Inc. 2012) formed a flexible docking platform for determining the binding affinity for salbutamol in wild and T164I variant [46]. In the docking process, receptor grid was generated around the co-crystallized established inverse agonist present in the agonist binding site in the β2AR structure. Potential of non-polar parts were softened by scaling van der Waals radii with a scaling factor of 1.0 at the cut off partial charge maintained at 0.25. Salbutamol was then docked at the generated grid containing active site. Extra precision (XP) docking was performed with flexibility involving sampling of nitrogen inversion and ring conformations with energy window of 2.5 Kcal/mol. Post-docking ligand-receptor complex was energy minimized with a default protocol setting of distance-dependent dielectric constant of 2.0 and maximum number of minimization steps of 100. Threshold for rejecting minimized pose was set to 0.5 Kcal/mol. At most 10,000 poses for salbutamol were collected and the best pose was selected by rejecting coulomb-vdW greater than 0.0 Kcal/mol. Further, duplicate poses were removed if RMSD was less than 0.5 Å and maximum displacement was less than 1.3 Å. The final binding affinity was determined by Glide Score or XPG (Extra Precision Glide) score with a threshold strain correction of 4.0 Kcal/mol.

### Molecular dynamic simulations

The complete molecular dynamic simulation for salbutamol in wild and T164I variant was performed using Desmond program version 3.7 (D.E. Shaw Research, NY, Maestro-Desmond Interoperability Tools, 2014) with OPLS 2005 force field algorithm [47] available in the Maestro interface of Schrödinger suite (Schrödinger, LLC, New York, NY, 2015. Inc. 2015). Prepared protein-ligand complexes were equilibrated in POPC lipid bilayer membrane (hydrophobic thickness of 31.6±1.3Å and a tilt angle of 7±1°) through importing in Desmond setup wizard and solvated in an orthorhombic periodic box of SPC water molecules and neutralized with counter ionic concentration of 19.109 mM having 15 chloride ions [48]. The system was subjected to the local energy minimization using a hybrid method of the steepest decent and limited-memory Broyden—Fletcher—Goldfarb—Shanno (LBFGS) algorithms [49] for maximum of 5000 steps until a gradient threshold (25 kcal/mol/Å) was reached. The simulation system was relaxed by constant NPT ensemble condition to generate data for post-simulation analyses. The temperature was defined at 300 K using Nose—Hoover thermostats [50] at stable atmospheric pressure (1 atm) maintained by Martyna—Tobias—Klein barostats [51]. The multi-time step reversible reference system propagation integrator algorithm (RESPA) was used to investigate the equation of motion in dynamics [52]. Atoms involved in hydrogen bond interaction were constrained by SHAKE algorithm [53]. The short range electrostatic and Lennard—Jones interactions were estimated by setting up the cut off value with radius of 9Å. The long-range electrostatic interactions were evaluated by particle mesh Ewald (PME) method using periodic boundary conditions [54, 55]. The final production of dynamics was carried for 10 ns and the trajectory potentials were analyzed by simulation event analysis available in Desmond module of Schrödinger suite.

Complete methods of molecular modeling were carried out on Dell precision work station configured with Intel (R) Xenon(R) 2 Duo CPU E7600 @ 3.06GHz processor with memory of 8 GB RAM running on LINUX operating system.

## Results

The current study is sought to identify molecular rationales of sub optimal binding of salbutamol in T164I variant in structural details employing integrated molecular modeling investigations.

Prior addressing the inefficiency of ligand receptor interaction in T164I variant, we first pursued to study the structural and functional implications of T164I variance in β2AR. The vulnerability of variation calculated by different mutation assessor modules predicted T164I variant to be benign or neutral (Table 1). Since these servers predict mutation leading to the disease states, the prediction actually holds acceptable for the T164I variation. In fact there are convincing reports suggesting lack of associating of T164I polymorphism with susceptibility to develop asthma or any other respiratory diseases in overall population. Meta-analysis of case-control studies by Contopoulos-Ioannidis *et al*. in 2005 showed insignificant odds ratios proving lack of association of T164I polymorphism with risk of developing asthma [56]. In addition, genetic association studies by Liang *et al*., in 2014 and Pagaria *et al*. in 2007 respectively in Chinese and British population also confirmed lack of association of ADRB2 polymorphisms with asthma incidence or susceptibility [57, 58]. In coherence, Migita *et al*. in 2004 also reported insignificant association of all the ADRB2 gene polymorphisms with a risk of asthma across the ethnic groups [59]. All these association studies therefore find convincing validation for benign prediction by mutation assessor modules.

**Table 1:** Prediction of vulnerability of T164I variation by computational programs and calculation of total structural deviation.

Prediction programs for disease related mutations	Score	Prediction	
SIFT	1	tolerated	
Polyphen2	0.008	benign	
PANTHER	0.114	neutral	
PhD-SNP	0.188	neutral	
SNAP	0.245	neutral	
Meta-SNP	0.326	neutral	
MAPP	85%	neutral	
Predict SNP	74%	neutral	
SDM	SASA prediction Wild = 58.3% T164I variant = 41.9%	Pseudo ΔΔ*G* = 2.79 Kcal/mol	Prediction: destabilizing and cause protein malfunction
STRUM	ΔΔ*G* = 1.47 Kcal/mol		
iMUTATNT3.0 Prediction Effect: Decrease in Stability, ΔΔ*G* = 1.23 Kcal/mol			
Total Structural RMSD (Å) = 5.63			

However the malfunctioning of the receptor cannot be ruled out as shown by Site Directed Mutator (SDM), I-Mutatnt 3.0 and Strum programs (Table 1). SDM predicted significant decrease in stability of the β2AR upon T164I substitution and also assigned malfunctioning status of the receptor. In addition, SDM predicted solvent accessible surface area (SASA) to reduce to 41.9% in T164I variant from 58.3% in wild receptor. The results of SDM are further supported by free energy calculation programs like Strum and iMutatnt 3.0 which in addition predicted the mutation to be destabilizing. In further approach the overall structural departure upon T164I variation was assessed by calculating the RMSD which revealed significantly higher value of 5.63 (Table 1).

From the observations deduced by mutation effect prediction programs it is worth noticing that, T164I may not necessarily form pathogenic variant, nevertheless, has its prominent role in structural perturbation of the receptor leading to disease modulation (but not disease causing) like for drug response.

**Fig 1:**
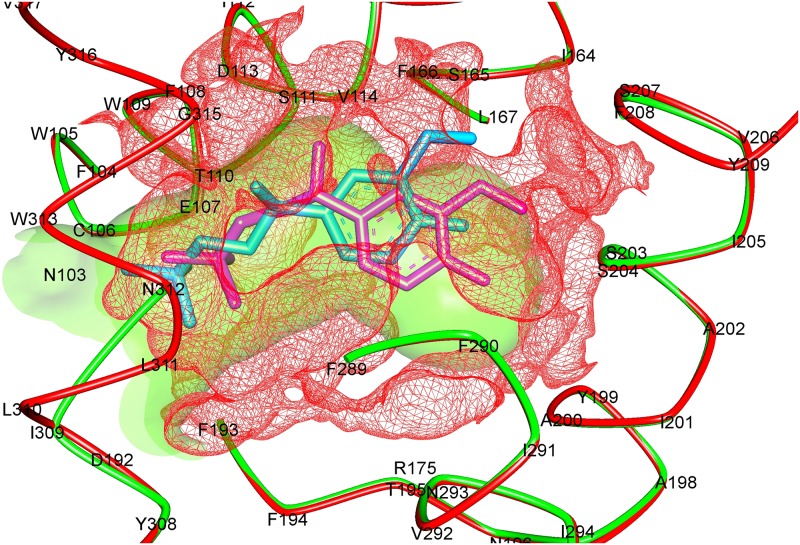
The overlapping cartoon depicts binding cavities of wild (green solid) and T164I (red mesh) β2AR. Volume of the cavity in wild β2AR is 404.48 Å^3^, upon substitution the cavity expands to 520.70 Å^3^. Poses of salbutamol (Sea green in wild, and pink in T164I variant) are shown in the binding cavity.

In the further step, the effect of structural distortions upon variation was analyzed at the agonist binding site. Calculation of volume and surface area of the active site of wild and T164I variant in fact revealed notable differences (Table 2). The volume of the active site increased from 404.48Å^3^ to 520.70 Å^3^ upon substitution followed by increase in the surface area by 21.3% (Fig 1). Also, the hydrogen bond donors, acceptors and hydrophobic residues in T164I were drastically reduced in T164I receptor. It may therefore be presumed that higher order of flexibilities at the active site may lead to gross conformational changes in the protein, and this also may explain the reduced binding of salbutamol in the T164I β2AR. However the binding analysis would throw better picture, for which we performed docking analysis and molecular dynamic simulations.

**Table 2:** Calculation of binding site properties of wild and T164I variant β2AR.

Binding site topology	Wild	T164I variant	T164I variant /Wild	Surface/Volume for Wild	Surface/ Volume for T164I variant
Volume [Å³]	404.48	520.70	1.28	1.67	1.60
Surface [Å²]	676.52	834.92	1.23		
Depth [Å]	22.72	32.67	1.43		
**Functional group descriptors (n)**					
Hydrogen bond donors	26	18			
Hydrogen bond acceptors	116	112			
Metals	0	0			
Hydrophobic interactions	114	82			

In the further approach, structural perturbation incurred upon T164I substitution in β2AR was analyzed in atomistic detail by performing OPLS force field aided molecular dynamic simulations for 10 ns. Simulation descriptors like RMSD, Cα Root Mean Square fluctuations (RMSF) of amino acid residues, Radius of gyration (rGyr) were calculated in reference to initial minimized structure as a function of time which facilitated to understand the dynamic effect of T164I variation.

**Fig 2:**
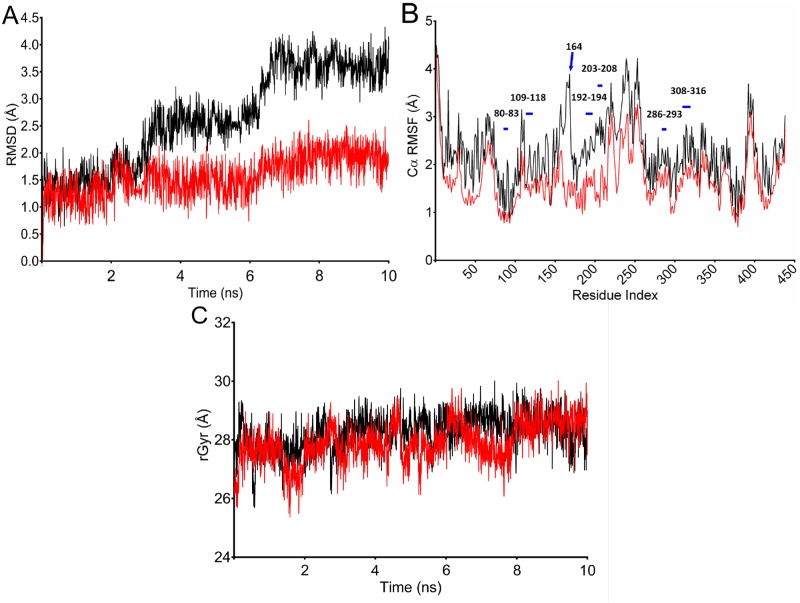
Structural perturbation incurred in β2AR upon T164I variation analyzed by simulations. (A) RMSD calculated for 10 ns of simulation trajectory for wild and T64I β2AR. β2AR is rendered with high conformational flexibility upon T164I substitution as observed from gradual rise in RMSD after 2.7 ns for T64I β2AR. (B) RMSF calculated for residues in wild and T164I variant. Purple bars indicate active site residues; arrow pointing the peak represents the site of variation. (C) Compactness of wild and T164I β2AR assessed by calculating radius of gyration. Trajectory lines for wild and T164I β2AR are represented in red and black color respectively.

From comparison of trajectories, a similar pattern of deviations of ~ 1.5 Å were observed up to 2.6 ns for both wild and T164I variant; however a trajectory showed gradual rise from 2.7 ns for T164Ile variant (Fig 2A). At this point of trajectory, the effect of variation can be well represented. Both wild and variant structure converged at 10 ns however with a significant difference in final RMSD values. The wild structure adopted least deviation corresponding to initial minimized conformation which converged at ~1.7 Å indicating wild system to be reasonably stable during the simulation. In contrast, the T164Ile variant structure converged at ~ 4.0 Å implying greater structural departure from the initial minimized structure. Although the simulation was programmed for 10 ns, 3 to 6 ns were sufficient to confirm divergence of T164Ile variant structure from wild structure suggesting substantial change in topology of β2AR upon T164I variation. Interestingly, the results of simulations were in assertion by the results obtained from free energy prediction by SDM, Strum I-Mutant 3.0 programs which also predicted the T164I variation to be destabilizing.

Further, Cα RMSF was calculated to interpret average fluctuations of each amino acid residue during simulation (Fig 2B). Values of residue RMSF was in coherence with observed RMSD- for both being higher for T164I variant receptor relative to the values in wild receptor. A keen perusal at the residue RMSF revealed highest deviation at 164^th^ variant position. Besides, the variation also impacts almost ten neighboring residues spanning across 154^th^ to 174^th^ position. It is however quite reasonable to have high RMSF at the site of variation (or for neighboring residues spanning variation), nevertheless, it was remarkable to note that even the residues at ligand binding site showed higher RMSF indicating the T164I substitution has an enormous impact on agonist binding site. Therefore, the overall results of RMSD followed by RMSF calculation may possibly authenticate an observation that unstable complex formation of salbutamol in T164I variant receptor should be an obvious phenomenon (due to high fluctuations of residues at the active site in the variant receptor) which in turn hints for suboptimal binding of salbutamol in T164I variant. In the following step, compactness of receptor upon variation was interpreted by recording radius of gyration, which showed no difference across the simulation trajectory, indicating the compactness of the protein is not necessarily altered upon variation (Fig 2C).

The major quest of the current study lies in predicting the binding efficiency of salbutamol in T164I variant. We therefore performed two analyses, one—ligand receptor docking which formed a static basis for interpreting the binding affinity and two—molecular dynamic simulations of ligand-receptor complex.

Employing Glide’s extra precision (XP) mode of docking, we observed that binding affinity (represented as a function of docking score or XP G (extra precision Glide) score) of salbutamol was -16.697 against wild β2AR while affinity reduced by two folds in T164I variant with a score of -7.803 (Table 3). Post docking analysis involving docking score fragmented into forces like evdw (van der Waals), ecoul (coulumbic or electrostatic forces) and in particular reduced XP HBond (extra precision hydrogen bonding efficiency) furthermore proved declined binding efficiency of salbutamol in the T164I variant. It is quite evident that salbutamol although being a high affinity agonist for adrenergic receptor at the wild state, nevertheless shows declined binding affinity by 2.13 folds for its T164I variant. In our previous investigations, we reported salbutamol refractoriness in asthmatics by recording lung function volumes (FEV_1_ reversibility). The FEV_1_ percentage reversibility was 10.14% in asthmatics recessive for Thr/Ile polymorphism while 25.55% in asthmatics with homozygous wild genotype (p value < 0.001). It is worth noticing that decline in the FEV_1_ percentage reversibility (or refractoriness to salbutamol) in homozygous recessive subjects can be due to reduced binding affinity of salbutamol in T164I variant as evident from docking studies performed herein.

**Table 3:** Energy descriptors determining the binding efficiency of salbutamol in wild and T164I variant.

Energy Descriptors	Wild	T164I variant
docking score (XP G Score)	-16.697	-7.803
glide evdw	-32.467	-30.086
glide ecoul	-13.720	-10.329
glide einternal	7.821	1.818
glide emodel	-61.985	-60.664
XP HBond	-4.699	-2.447
glide ligand efficiency	-0.629	-0.535
glide ligand efficiency sa	-1.618	-1.375
glide ligand efficiency ln	-2.791	-2.371

**Fig 3:**
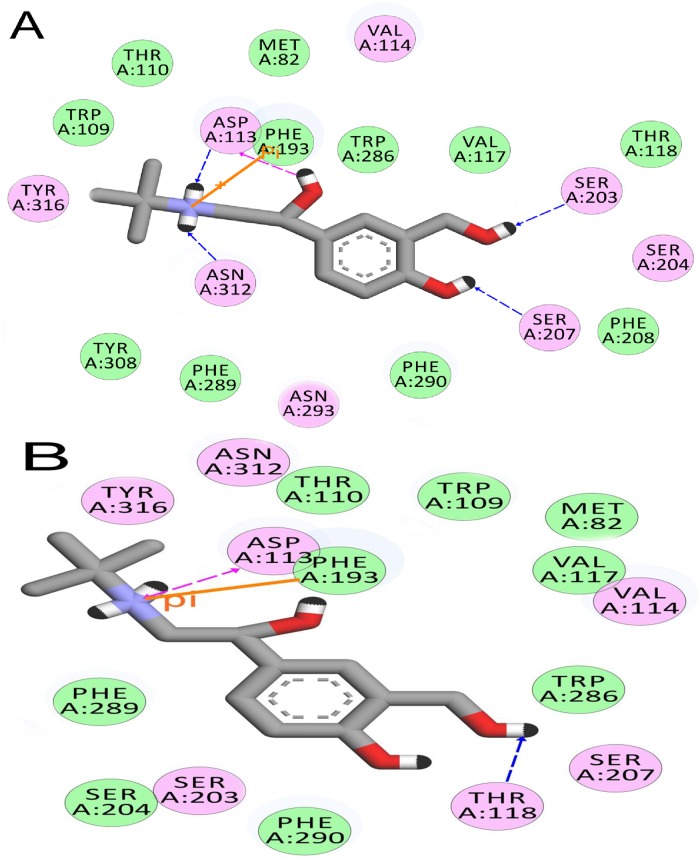
Molecular interaction diagrams of salbutamol deduced from molecular docking. (A) Salbutamol in Wild and (B) Salbutamol in T164I β2AR. Residues in green participate in van der Waals interaction, residues in pink form electrostatic interactions with the salbutamol. Hydrogen bonds are shown as blue (acceptor) and pink (donor) arrows. Pi-pi interactions are shown with orange solid line.

**Fig 4:**
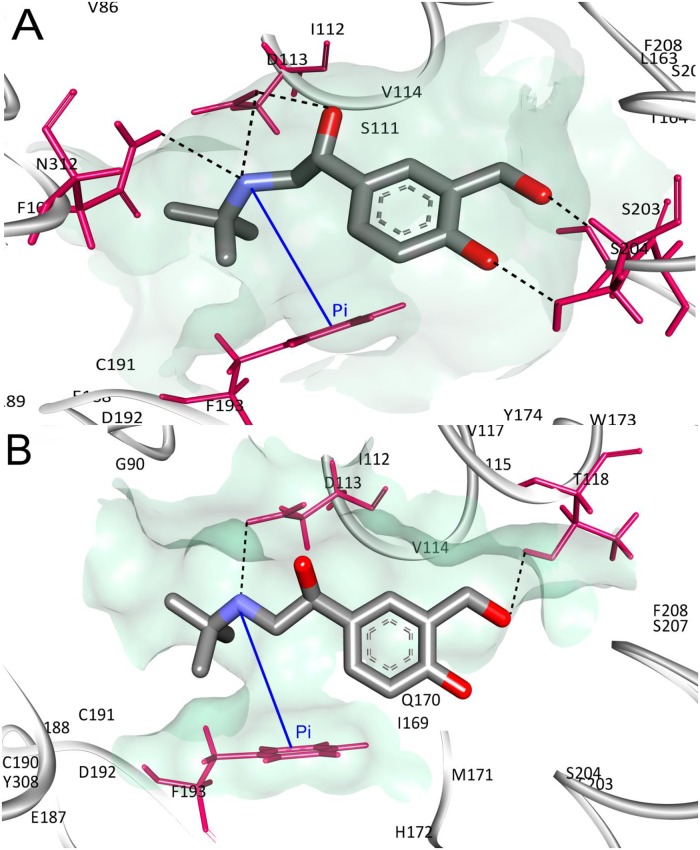
Interactions of Salbutamol docked in the agonist binding site. H bond and pi-pi networks of salbutamol in (A) wild and (B) T164I β2AR. Black discontinuous lines are H bonds, blue line represent pi-pi interactions.

The interaction profile of salbutamol in wild and T164I variant receptors is consolidated in Table 4. Close perusal reveals hydrogen bonding efficiency of salbutamol is drastically reduced in T164I variant receptor (5 and 2 hydrogen bonds in wild and T164I variant receptor respectively) which is also reflected from reduced value of XP HBond descriptor shown in Table 3. In addition, number of residues interacting with electrostatic and van der Waals forces with salbutamol was considerably few in T164I variant compared to wild receptor (Figs 3 and 4). Therefore, these contacts and especially decline in the hydrogen bonds form the principal basis for reduced affinity of salbutamol in the T164I variant.

**Table 4:** Interaction profile of salbutamol in wild and T164I variant of β2AR.

	Wild		T164I variant	
	# of contacts	Interacting Residues	# of contacts	Interacting Residues
H bonds	5	2(Asp 113), Asn 312, Ser 203, Ser 207	2	Asp 113, Thr 118
pi-pi stacking	1	Phe 193	1	Phe193
Salt bridges	0		0	
Electrostatic contacts	8	Asp 113,Val 114, Tyr 316, Asn 312, Ser 203, Ser 204, Ser 207, Asn 293	7	Asp 113,Val 114, Thr 118, Ser 203, Ser 207, Asn 312, Tyr 316
van der Waal contacts	11	Met 82, Trp 109, Thr 110, Val 117, Thr 118, Phe 193, Phe 208, Trp 286, Phe 289, Phe 290, Tyr 308	9	Met 82, Thr 110, Phe 193, Trp 109, Phe 289, Phe 290, Ser 204, Trp 286, Val 117

To validate the credibility of docking results, two independent ligand-receptor simulations for 10 ns were performed; one with salbutamol in complex with wild receptor and another in complex with its T164I variant. As anticipated, in course of simulation, salbutamol in complex with wild receptor demonstrated higher stability relative to salbutamol in T164I variant (Table 5). Evident from the average energy scores, the stability in terms of total free energy of simulation, salbutamol in the wild receptor was ~2.05 folds more stable than in complex with T164I variant testifying reduced binding affinity of salbutamol in the T164I variant.

**Table 5:** Energy analysis of ligand-receptor complex over simulation trajectory.

	Wild			T164I variant		
	Avg	Std.dev	Slope (ns ^-1^)	Avg	Std.dev	Slope (ns ^-1^)
Total energy (Kcal/mol)	-62982.07	238.54	0.17	-32708.99	470.157	0.148
Potential Energy (Kcal/mol)	-76269.83	1450.37	0.07	-43433.0	1110.216	0.033
Volume (A^3^)	38489.35	35.93	-0.27	38510.29	27.929	-0.205
Degrees of freedom	139484			140196		
Particles	64921			65077		
Atoms	64921			65077		
**Standards**						
Ensemble	NPT					
Duration (ns)	10					
Temperature (K)	300					
Pressure(bar)	1					

**Fig 5:**
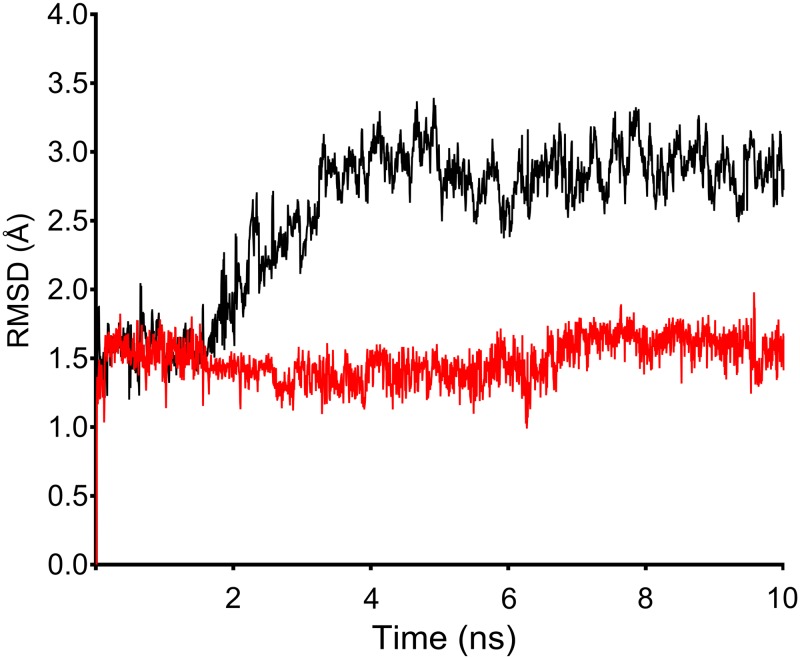
“Ligand fit in protein” RMSD for salbutamol in wild (red) and T164I β2AR (black) projected at a simulation trajectory of 10 ns.

A comparative analysis of “ligand fit in protein” RMSD trajectories for salbutamol in wild and T164I variant β2AR revealed massive differences (Fig 5). “Ligand fit in protein” RMSD calculates how well the ligand fits in an active site during simulation. For the first 1.8 ns of simulation, similar RMSD in both wild and T164I variant was observed, however, there was a steep rise after 2.3 ns for T164I variant receptor. Besides this, it is interesting to note that throughout the simulation time there are no stable RMSD observed in T164I variant indicating transient and non—favourable interactions of salbutamol in T164I variant when compared to wild receptor with stable and small deviations. At the final time step of 10 ns, RMSD converged at a stable RMSD of 1.5 Å for wild receptor-salbutamol complex while significantly higher RMSD of 3.1 Å was observed for T164I-salbutamol complex. Therefore overall simulation analysis suggested unstable interactions of salbutamol leading to reduced affinity for T164I β2AR.

**Fig 6:**
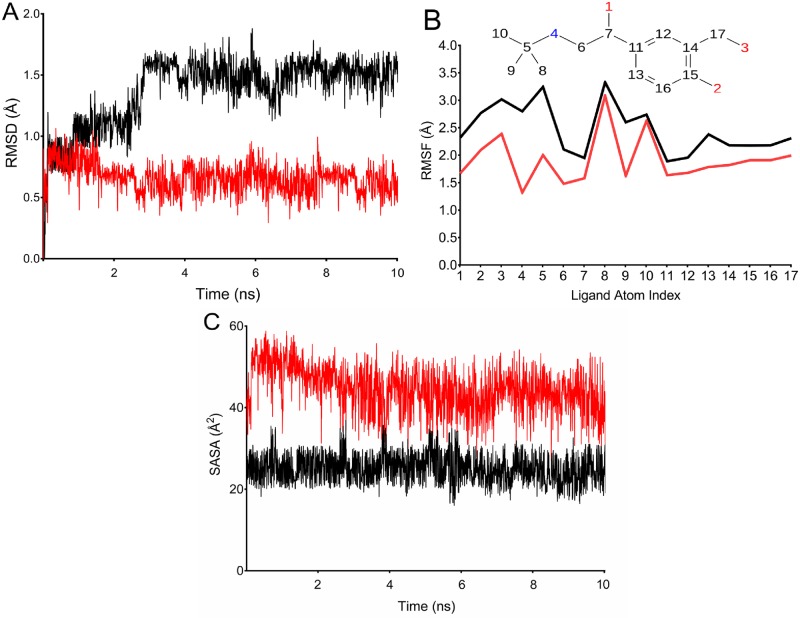
Trajectory analysis of salbutamol in complex with wild and T164I β2AR. (A) RMSD of salbutamol with respect to the reference conformation in wild and T164I β2AR. (B) ‘Fit on protein' line shows atomic fluctuations (RMSF) with respect to the receptor. Corresponding atoms of salbutamol is shown as 2D structure in the top panel. (C) Solvent accessible surface area of salbutamol in course of simulation. Trajectory lines for wild and T164I β2AR are represented in red and black color respectively.

Subsequent calculation of dynamic properties of salbutamol in wild and T164I variant in addition exemplified the unstable complex formation of salbutamol in T164I receptor. As shown in Fig 6A, ligand deviations (L-RMSD) for salbutamol was stable for first 1.5 ns, however a steep increase in deviation finally reaching 2.4Å can be observed for T164I receptor while stable deviations with less than 1 Å was recorded for salbutamol in wild receptor. The calculations of ligand RMSD is clearly supported by Ligand Root Mean Square Fluctuation (L-RMSF) (Fig 6B). L-RMSF provides insights on deviations of ligand atoms and the way each ligand atom interacts with the protein with their binding entropy in course of simulation event. As depicted in Fig 6B, salbutamol displays higher degree of fluctuations in T164I receptor than being in wild receptor. An important observation which is worthy to mention that, during simulation, oxygen (atom positions1, 2, 3) and nitrogen (atom position 4) of salbutamol which majorly are hydrogen bond donors/acceptor atoms, significantly displayed higher degree of fluctuation. Higher atomic RMSF therefore evidently suggested declined propensity of salbutamol to form hydrogen bonds in T164I variant and hence in turn accounts to higher binding entropy or less stability of salbutamol in complex with the T164I variant. Furthermore in support to the above observations considerable reduction in the solvent accessible surface area was also observed for salbutamol in T164I variant (Fig 6C) which additionally proves its declined contacts and reduced stability in T164I β2AR.

**Fig 7:**
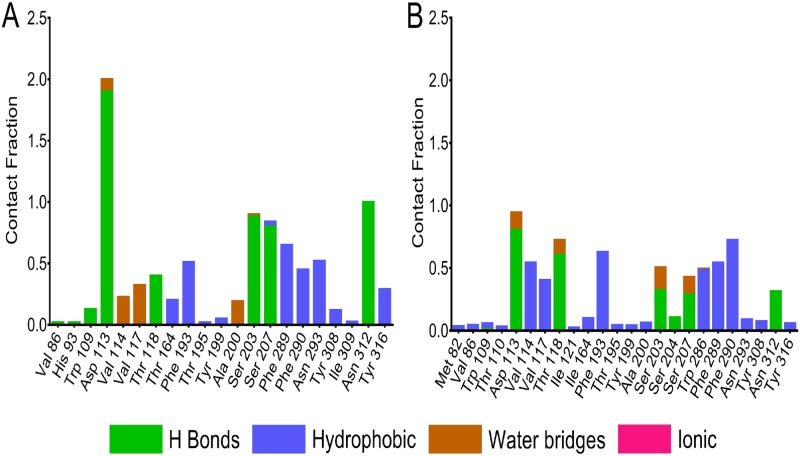
Contact fractions of ligand-receptor interactions in course of simulation. Simulation interaction diagram showing contact fractions of residues interacting with salbutamol in (A) wild and (B) T164I variant of β2AR.

In the further process, the residues contacts over the trajectory were monitored throughout the simulation process. Protein-ligand interactions were categorized into four major types: hydrogen bonds, hydrophobic, ionic and water bridges. A comparative simulation interaction diagram for salbutamol in wild and T164I variant is shown in Fig 7A & 7B. Interaction fraction of each residue differs in both complexes. A significant difference can be observed with the fraction of H-bonds formed between salbutamol in wild and T164I variant. The fraction of H-bond is considerably higher in wild receptor. The hydrogen bonding residues common in wild and T164I variant are Asp 113, Thr 118, Ser 203 Ser 207 and Asn 312 however interaction fractions are comparatively reduced in T164I variant. It can be noted that H bond fraction for Asp 113 in wild is 1.8 which is quite above the maximum fraction of 1.0, implying two H bonds are maintained between Asp 113 and salbutamol for over 90% of the simulation time. In T164I variant however H-bonding fraction of Asp 113 is 0.8 which is nearly two folds reduced than in the case of wild receptor. In addition to this, the hydrogen bond fraction of Ser 203, Ser 207 and Asn 312 in wild receptor is significantly higher than in T164I receptor. Trp 109 is yet another H bond donor to salbutamol in the wild receptor which is completely absent in T164I variant. Contrastingly, the H bond fraction of Thr 118 is slightly higher in the T164I variant; also H bond fraction of Ser 204 is observed in T164I variant which otherwise absent in the case of wild receptor. The minor increase in the H bond fractions provides “*transient stability*” for salbutamol in T164I receptor. Additionally, throughout the simulation trajectory, hydrophobic and water contact fractions were considerably reduced in T164I variant.

**Fig 8:**
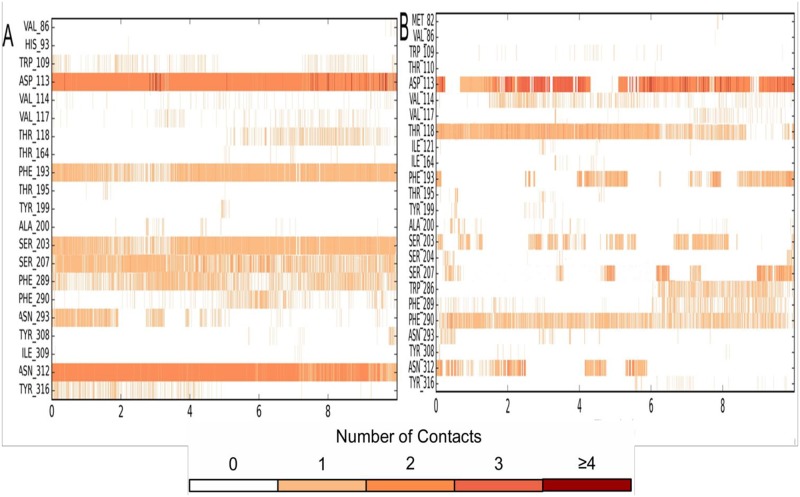
Timeline representation of ligand—Receptor interactions. Residues interacting (all the interactions including H-bonds, Hydrophobic, Ionic, Water bridges) with salbutamol in (A) wild and (B) T164I variant of β2AR.

The time-line contacts of salbutamol in wild (Fig 8A) and T164I variant (Fig 8B) across 10 ns of simulation trajectory supports the interaction fractions diagrams as mentioned in Fig 7. The time line contacts for all important residues participating in H bond interactions with salbutamol were recorded to have interrupted contacts in T164I variant. For instance, Asp 113, Asn 312, Ser 203 and Ser 207 have high interrupted contacts, in addition, at the time steps of available contacts, these residues show less than 2 contacts which further demonstrates declined propensity of salbutamol to form stable ligand-receptor complex with T164I β2AR. However, transient stability of salbutamol in T164I β2AR which is brought about by Ser 204 and Thr118 as shown in Fig 7B is also reflected in time line contacts in Fig 8B.

**Fig 9:**
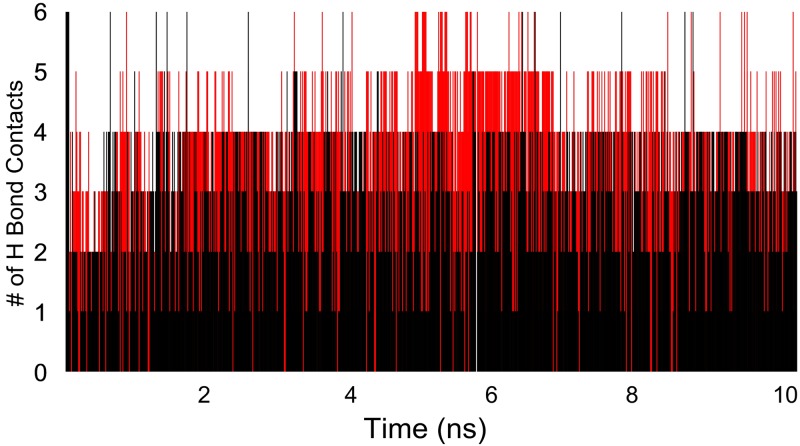
Number of H bonds contacts established by salbutamol with wild (red) and T164I β2AR (black) in each frame of simulation. Reduced H bond contacts for T164I variant can be observed.

The hydrogen bonding efficiencies of salbutamol in both the receptors were evaluated by calculating number of contacts in each frame of the simulation (Fig 9). Approximately 2 folds declined H bond was recorded for salbutamol in T164I receptor than in wild receptor which expounds higher deviations and reduced affinity of salbutamol in T164I β2AR variant.

**Fig 10:**
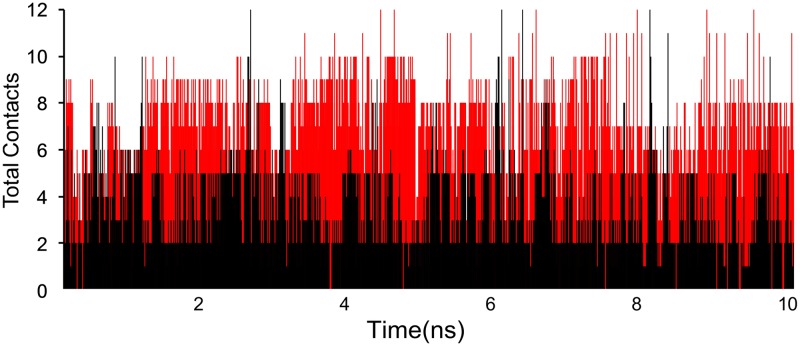
Total contacts (H-bonds, Hydrophobic, Ionic, Water bridges) of salbutamol with wild (red) and T164I (black) β2AR recorded at each frame of simulation. Declined contacts of salbutamol in T164I variant are apparent.

A timeline representation of total contacts (including H-bonds, Hydrophobic, Ionic, Water bridges) over the course of the simulation is depicted in Fig 10. It is quite evident that the total number of contacts rarely reaches 12 for salbutamol in T164I variant which yet again proves reduced affinity and inept propensity of salbutamol to form stable complex with T164I variant.

Altogether, the results presented herein provide dynamic insights of sub-optimal binding of salbutamol in the T164I variant in atomic details through exhaustive computational modeling approaches.

## Discussion

The profound effect of inefficient binding of agonists to T164I variant has been established for past two decades put forth by pioneering investigation by Green and co workers [34]. This investigation involved membrane competition studies in the presence of guanine nucleotide showed nearly four folds reduced ligand binding affinity upon stimulation by agonists and 50% decline in adenyl cyclase activity in recombinant CHW cells expressing T164I recessive allele. An independent investigation led by Green himself in 2001 however used broader spectrum of agonists to evaluate their binding affinity against T164I variant [60]. The study involved all common established β2 agonists including isoproterenol, salbutamol, metaproterenol, terbutaline, formoterol, and salmeterol and interestingly all of them displayed three folds decreased binding affinities with significant impairment of maximal stimulation of adenylyl cyclase. The reduced affinity in T164I receptor was in addition ascertained from agonist binding studies which resulted in low binding affinity constants and monophasic curves along with depressed agonist-stimulated adenylyl cyclase activity which substantially confirms inefficient binding of agonists in T164I receptor [61].

These remarkable studies in fact have been successful in establishing T164I substitution as an important pharmacogenetic locus for the commonly administered agonists in respiratory diseases.

Therefore, in view of these studies as a reference, we therefore hypothesized that reduced affinity of agonists should be the most probable cause for refractoriness in patients those harboring T164I variant. We in our previous studies in Indian asthmatics reported significant association of T164I polymorphism with salbutamol refractoriness in asthmatics [36]. In coherence to our study, similar results were also reported in Danish population attributing T164I polymorphism to salbutamol refractoriness [62]. It was therefore most likely that the reduced affinity of agonist to T164I variant as mentioned by Green *et al* may therefore possibly explain observed “salbutamol refractoriness” in patients homozygous for the variant (refractoriness manifested as declined lung function volumes). To ensure that the phenomenon of “reduced binding of salbutamol in T164I variant” was an underlying principle for refractoriness in patients; we have therefore pursued the present investigation to address this phenomena at structural grounds through integrated computational approaches.

In the first step, vulnerability of variation was assessed by mutation effect prediction programs which showed the variant to be benign. These programs predict the effect of mutation at the planes of disease associations and given good number of meta-analysis reports have shown lack of significant association with disease outcomes in any ethnic population [63, 64], the benign prediction actually holds validated.

Although T164I may not form susceptible variant to cause any disease, nevertheless its immense role in modulating the disease condition like that of altering the drug response cannot be ruled out. In further investigations we found that the stability of the receptor was grossly decreased and predicted to be malfunctioning as shown by statistical potential energy functions employed in programs like iMutant 3.0, Strum and SDM. The structural departures upon substitution were assessed by comparative analysis of binding cavity of the receptors and calculating total root mean square deviations of all atoms of the receptor. A gross modification in the active site was evident from relative increase in the volume and surface area in the T164I receptor which proved structural perturbations incurring upon substitution. In addition to this, total structural RMSD of 5.2 also testified significant structural changes to take place upon variation.

The change in structural configurations due T164I variation was however more obvious when molecular dynamic simulations were performed. For a simulation trajectory of 10 ns, the converged RMSD for T164I variant was 2 folds higher than wild β2AR suggesting significant structural departure to incur upon variation. The results of RMSD was supported by residue wise RMSF which showed amino acids at active sites had higher fluctuations throughout the simulation and this perhaps may explain the reduced binding affinity of salbutamol in the T164I β2AR. In order to substantiate suboptimal affinity of salbutamol for T164I variant, docking and ligand-receptor complex simulations were carried out. Docking studies showed that salbutamol although being a high affinity agonist for adrenergic receptor at the wild states, showed decline binding affinity by 2.13 folds for T164I variant. This was more apparent when simulations were performed. At the final time step of 10 ns, converged RMSD for salbutamol in complex with T164I β2AR receptor was 1.88 folds higher than salbutamol in complex with wild receptor, therefore, clearly implying salbutamol to be highly unstable in T164I variant. In addition, when ligand deviations were recorded, salbutamol showed considerably higher deviations along with reduced surface contacts with T164I variant confirming less propensity of salbutamol to form stable complex with T164I β2AR. Furthermore, a close perusal of ligand-receptor interactions across the simulations revealed drastic decline in residue contacts of T164I variant with salbutamol. Especially fraction of hydrogen bond and hydrophobic interactions were considerably reduced in salbutamol- T164I β2AR complex which further illustrates the rationale behind suboptimal binding of salbutamol in the T164I variant.

β2 agonists and especially salbutamol forms the first line of therapy for emergent management of airway constrictions. Unfortunately, patients harboring T164I β2AR variant are to a large extent refractory and barely benefit out of to β2 agonists therapies. It therefore necessitates for current disease management protocols to establish pharmacogenetic methods of treatment with special consideration for patients with T164I β2AR variant. In our previous studies (involving computational studies and patients follow up studies) we found that fenoterol to have consistent binding affinity in wild and T164I β2AR [65]. Although being consistent, fenoterol was not an efficient binding candidate as salbutamol in wild state of the receptor. In such case, the treatment strategy would subsequently rely on salbutamol for patients with wild β2AR and fenoterol for the patients bearing the variant. Such selective administration of agonist can be thought of; nonetheless, it should be cumbersome process as it mandates screening of patients for mutation and then prescribed suitable β2 agonist. The diligent and logical method would otherwise be to virtually screen and identify a promiscuous β2 agonist which must be bestowed with high affinity for both wild and T164I β2AR. Such promiscuous agonist could be thought to overcome the binding deficiencies of salbutamol in T164I variant and fenoterol in wild receptor as well.

## Conclusion

Altered interactions of agonists in T164I β2AR has been known for long, however, structural basis of binding affinity were still elusive. We have therefore proposed and put forth structural rationales and dynamic aspects of sub optimal binding of agonist like salbutamol in T164I β2AR in very atomic detail through robust computational methods. We anticipate the investigations presented herein will pave way for designing rational agonists targeting T164I β2AR for the better management of respiratory diseases in the near future.
